# Preparation and pharmacokinetic evaluation of a sertraline-methylpropyphenazone prodrug: a comparative metabolic study on the plasma and brain tissues of rats using LC-MS/MS analysis†

**DOI:** 10.1039/d4ra08357a

**Published:** 2025-01-28

**Authors:** Alaa Khedr, Alanoud Ali, Tarek S. Ibrahim, Ahmed K. Kammoun

**Affiliations:** a Department of Pharmaceutical Chemistry, Faculty of Pharmacy, King Abdulaziz University P.O. Box 80260 Jeddah 21589 Saudi Arabia akhedr@kau.edu.sa +966 553399718

## Abstract

A mutual prodrug of sertraline-methylpropyphenazone (SER-MP) was prepared and characterized using a spectral method. The yield of the prepared SER-MP was 90%, and its purity reached 98.8%. The metabolic fate of the prepared SER-MP *versus* sertraline (SER) was investigated in the plasma and brain tissues of rats. A solid-phase extraction procedure was developed and validated for the optimal recovery of SER, 3-hydroxymethylpropyphenazone (3-OHMP), and SER-MP from plasma and brain tissues. The extraction efficiency for the targeted analytes was improved from 93.5% to 98.0% using Chromabond® C8-100 mg solid-phase extraction columns. A high-performance liquid chromatography-triple-quad-mass spectrometric method was developed and validated to quantify the SER, SER-MP, and potential metabolites. The pharmacokinetic parameters showed that the time to reach the maximum plasma concentration (*t*_max_) for both SER and SER-MP was 6 hours, and the maximum plasma concentration of SER-MP reached 192 ng mL^−1^ with an elimination half-life time of 50 hours. The plasma level of SER, which was released as a metabolite of orally administered SER-MP, was increased by 2.4 times compared to SER HCl administered at equimolar doses. The concentration of SER-MP in the rat brains remained approximately stable at 100 ng g^−1^ for 0.5 to 192 hours. The serotonin level in the rat brain homogenate was 50 to 90 ng g^−1^ for both the group receiving SER and that receiving an equal molar dose of SER-MP. This observation was consistent during a time range of 1 to 192 h from oral administration. Thus, this approach could lead to the development of more efficient antidepressant therapies with reduced side effects. The findings indicate that SER-MP could minimize serotonin syndrome risks by maintaining steady serotonin levels in the brain, thus improving patient safety and compliance.

## Abstract 2

A mutual prodrug of sertraline-methylpropyphenazone (SER-MP) was prepared and characterized using a spectral method.

## Graphical abstract

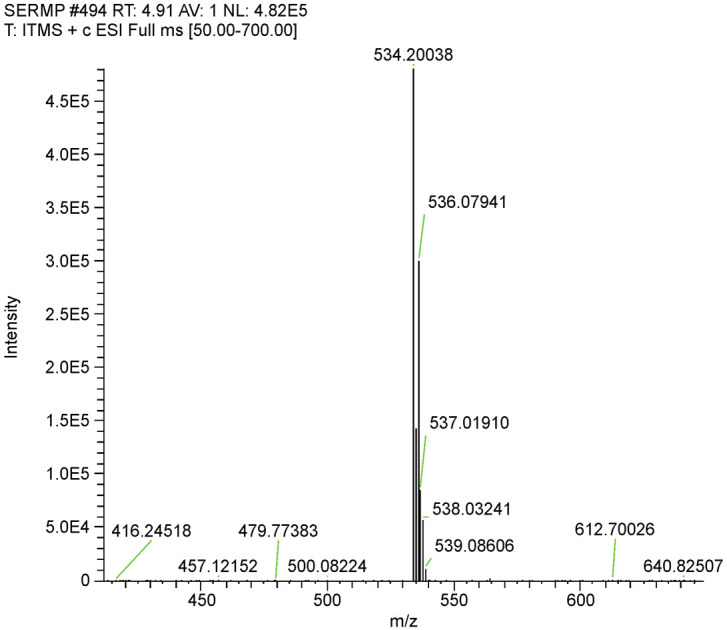

## Introduction

Sertraline hydrochloride, (1*S*,4*S*)-4-(3,4-dichlorophenyl)-*N*-methyl-1,2,3,4-tetrahydronaphthalen-1-amine, is a selective serotonin reuptake inhibitor (SSRI) that is commonly prescribed to treat major depressive disorder, social anxiety disorder, and many other psychiatric conditions.^1^ It is sold under the brand name Zoloft® in 25, 50, and 100 mg per tablet and oral solution forms as sertraline HCl.^2^ The mechanism of action of sertraline (SER) is presumed to be linked to its inhibition of CNS neuronal uptake of serotonin (5-hydroxytryptamine, 5HT), thereby increasing the serotonergic activity and serotonin synaptic concentration in the CNS.^3^ SER is taken orally and subjected to extensive first-pass metabolism, which means the liver rapidly metabolizes it before it reaches systemic circulation.^2^ SER is also slowly absorbed and reaches its highest concentration in blood plasma within 4.5 to 8.4 hours post administration.^4^ The half-life (*t*_1/2_) of SER is 25–26 h, while that for *N*-desmethyl-SER (metabolite) ranges from 66–80 h.^5,6^ About 98% of SER in plasma is bound to proteins, and the volume distribution of SER is approximately 20–25 L kg^−1^.^6^ About 40% of SER is excreted as metabolites in both cases.^4^ The remaining 12–14% of administered SER is eliminated in the urine as such.^3,7,8^*N*-Desmethyl-sertraline is a weakly active metabolite that attains a higher plasma concentration than its parent drug sertraline at a steady state.^2^ Other metabolic routes produce sertraline carbamic acid, *N*-hydroxy-sertraline, and the deaminated ketone of SER.^5^

The potential adverse effects of SER have been well documented and include gastrointestinal problems, such as nausea and diarrhea, difficulty sleeping, headaches, dizziness, sexual dysfunction in males, and a condition known as serotonin syndrome.^7–10^ These side effects pose a significant health concern for patients, leading to a burden on their well-being.

Various analytical methods have been reported to determine SER and its metabolites in bulk and biological matrices,^11^ including high-performance liquid chromatography (HPLC),^12–14^ capillary electrophoresis,^15^ gas chromatography (GC),^16^ and LC-MS/MS.^17,18^ Diverse extraction methods, such as liquid–liquid and solid-phase extraction, have been implemented to extract SER from its biological fluid before instrumental analysis, yielding recovery rates of 81.3% to 90.2%.^16,17^ The reported detection limits using HPLC-UV and GC-MS methods were 5.0 and 1.0 ng mL^−1^ plasma, respectively.^6,18^ In 1907, Meister Lucius and Brüning in Höchst synthesized 3-bromomethylpropyphenazone (BMP), [5-(bromomethyl)-4-isopropy-1-methyl-2-phenyl-1*H*-pyrazol-3(2*H*)-one], from propyphenazone and bromine.^19^ Several researchers have since investigated BMP as a coupling moiety for preparing prodrugs to improve the physiochemical properties or overcome some side effects. Sheha *et al.* prepared a methylpropyphenazone (MP) derivative of naproxen to reduce its side effects and metabolic fate.^20^ Mohamed *et al.* used propyphenazone as a prodrug with selective cyclooxygenase-2 inhibitors to improve and overcome the side effects of non-steroidal anti-inflammatory drugs.^21^ Khedr *et al.* used BMP as a derivatization reagent for the HPLC determination of ephedrine in urine with UV detection.^19^ These reports showed that propyphenazone-based prodrugs may enhance drug delivery and reduce the gastrointestinal side effects associated with acidic NSAIDs.^20,21^ Specifically, by temporarily masking the free acid group of NSAIDs, like ibuprofen or diclofenac, propyphenazone prodrugs can decrease direct contact between the acidic drug and the gastric mucosa. In addition, the propyphenazone moiety may also alter the conjugated drug's pharmacokinetics, potentially extending its duration of action or improving its distribution to target tissues.

The study hypothesizes that the development of a sertraline-methylpropyphenazone (SER-MP) prodrug could enhance the pharmacokinetic properties of sertraline, thereby improving its efficacy and safety. To test this hypothesis, the primary objectives of the present study were to synthesize and characterize the SER-MP prodrug, evaluate its metabolic fate compared to sertraline in rat models, and assess its pharmacokinetic parameters using LC-MS/MS analysis. By achieving these objectives, the study aimed to demonstrate that the SER-MP prodrug can offer a more effective and safer alternative for treating psychiatric conditions, potentially reducing the adverse effects associated with the present commonly used drugs and enhancing patient compliance.

## Experimental

### Chemicals

Sertraline hydrochloride >98.0% was purchased from the Tokyo Chemical Industry (TCI), India. Sildenafil, ≥98.0%, was purchased from Sigma-Aldrich GmbH (Steinheim, Germany). BMP was synthesized from methylpropyphenazone and bromine according to a method reported by Meister, Lucius, and Brüning in Höchst, 1907, with some modifications to improve the yield and purity to >95.0%.^19^ The methanol, formic acid, ammonia, and other chemicals used in the experiments were HPLC grade.

### LC-MS/MS conditions

An Agilent 6460 liquid chromatography – triple quad mass spectrometer (LC-QqQ-MS; Agilent Technologies, USA) was used for the qualitative and quantitative testing. Sildenafil was used as an internal standard (IS). The LC-MS system was controlled using MassHunter software (version B.03.01, Build 3.1.346.0). The MS conditions were set as follows: the ESI temperature was 330 °C, nitrogen gas flow rate was 11 L min^−1^, nebulizer pressure was 35 psi, and capillary voltage was 4000 V. The MS settings were optimized for each compound, including the fragmentor voltage, dwell time (sec), and collision energy voltage. The chromatographic separation was achieved utilizing an Eclipse-C18 column (100 × 4.6 mm, 3.5 μm particle size) connected to a Zorbax Stable Bond C8 (7 μm, 9.4 × 15 mm) Guard Cartridge (Agilent, Palo Alto, CA, USA).

A gradient elution program was applied using a mobile phase composed of methanol (solvent A) and 0.7 g L^−1^ ammonium acetate trihydrate in water with 0.1% formic acid (solvent B). The gradient program was configured as follows: 68% A from 0 to 2 min, raised to 96% A at 4.0 min, sustained at 96% A until 13 min, and then reverting back to 68% A at 13.1 min. The mobile phase was pumped at a flow rate of 0.4 mL min^−1^. The equilibration duration was 7.0 min. The sample injection volume was 5 μL. The total running time for the analysis was 20 min.

### Preparation of sertraline-methylpropyphenazone

Equal molar quantities of sertraline HCl (50 mg) and 3-bromomethylpropyphenazone (46 mg) were weighed and combined in a test tube. The mixed powder was then mixed with 5 mL acetonitrile, dissolved, and sonicated for 2 min. The solution was then transferred to a 250 mL round-bottomed flask and diluted with 20 mL acetonitrile and 25 mL chloroform. Approximately 100 mg anhydrous potassium carbonate was added, and the reaction mixture was stirred at room temperature for 24 h. The reaction progress was monitored using LC/MS-UV (254 nm), applying the positive ESI-scan mode, *m*/*z* 50–700.

The oily reaction product SER-MP was purified by column chromatography over silica gel, a mobile phase, dichloromethane (DCM), and then 9 : 1 DCM/ethyl acetate. The yield after purification was 91% w/w. The purity reached 98.8%, as determined by HPLC-DAD at 254 nm. The purified SER-MP was further used for the analytical studies and animal dosing.

### Preparation of 3-hydroxymethylpropyphenazone

3-Hydroxymethylpropyphenazone (HO-MP) was first identified as a metabolic product derived from isopropylantipyrine,^22^ with the researchers applying an optimized previously reported method.^20^ A working standard HO-MP solution was prepared from BMP to be used as a calibrant solution, and the modified preparation method was as follows: around 200 mg BMP was transferred to a 150 mL round-bottomed flask fitted with an air condenser and mixed with 20 mL water and approximately 25 mg sodium bicarbonate. After swirling, the mixture was heated over a boiling water bath on a hot plate at 110 °C and stirred for 2 h. The solution was cooled at room temperature, and then 25 mL CHCl_3_ was added, and the mixture was swirled for 5 min. The entire contents of the flask were then transferred to a 250 mL separatory funnel, mixed with 50 mL CHCl_3_, stoppered, shacked, and the organic layer was then separated and washed twice with water. The organic layer was collected in a 250 mL glass beaker. About 5 g anhydrous sodium sulfate was added to the organic layer and swirled to remove water. The clear supernatant was then transferred to a 150 mL round-bottomed flask and evaporated to dryness using a rotary evaporator under vacuum at 50 °C. The white residue was scratched off, collected in a 50 mL glass vial, and stored under vacuum over silica gel in a desiccator for five days. The purity of the product was verified using HPLC-UV at 254 nm and confirmed by LC-MS applying the positive ESI-scan mode, *m*/*z* 50–500. The precursor [M + H]^+^ was observed at *m*/*z* 247.

### Standard solutions

Separate standard solutions of 3-OHMP, SER HCl, SER-MP, and sildenafil citrate (IS) were prepared in methanol, each with a 1 mg mL^−1^ concentration. The working solutions were prepared separately for obtaining a 100 μg mL^−1^ concentration. Calibration standards were generated from the working solution by spiking control plasma (100 μL) with a standard mixture to get the final concentrations: 25, 100, 250, 500, 1000, and 2000 ng mL^−1^ plasma of each analyte. Three quality control (QC) samples were prepared, representing low (25 ng mL^−1^), medium (500 ng mL^−1^), and high (2000 ng mL^−1^) levels of each analyte.

### Sample preparation

Solid-phase extraction (SPE) was performed on the calibration standards prepared in plasma, quality control (QC) samples, rat plasma, and brain homogenate samples using Chromabond ® C8-100 mg columns (Macherey Nagel, Düren, Germany). The plasma and brain samples were thawed at room temperature and vortexed before use. The SPE column was conditioned with 1 mL methanol and 2 mL of 0.1% aqueous ammonia solution. The SPE column was filled with 25 μL of 0.1% aqueous ammonia solution, 100 μL plasma or brain homogenate, and 50 μL IS (20 ng μL^−1^). The SPE column was gently clicked to mix the solution, then allowed to elute just below the stationary phase surface before being trapped for 1 min in the stationary phase. The column was then washed with 1 mL of 0.1% ammonia solution, forcing nitrogen to eject water from the SPE column. The column was connected to a new port *via* a clean test tube, and the analytes were eluted using 3 mL ethyl acetate: *n*-hexane (2 : 1, v/v) and 1 mL methanol. The eluant was dried with nitrogen gas at room temperature, then reconstituted in 300 μL methanol, vortex, and transferred to a 0.9 mL total recovery autosampler vial, and dried with nitrogen gas. The residue was dissolved in 50 μL methanol, and a 5 μL volume was injected for LC-MS/MS analysis.

### Method validation

An improved solid-phase extraction approach and sensitive LC-MS/MS method were developed and validated to accurately quantify SER, SER-MP, 3-OHMP, and the main metabolites in rat plasma and brain homogenate. The selectivity, sensitivity, linearity, extraction efficiency, stability, accuracy, and precision were assessed following ICH guidelines for bioanalytical method validation and study sample analysis.^23^

The specificity was evaluated by analyzing blank samples with SER, 3-OHMP, and SER-MP. The linearity of the targeted analytes was evaluated at six concentration levels spanning the range of 25–2000 ng mL^−1^, and the calibration parameters were calculated. The method's accuracy was determined by calculating the estimated value for each QC sample compared with the actual concentration. The precision was assessed by analysis of QC samples at different concentrations (25.0, 500.0, and 2000.0 ng mL^−1^) on separate days. The lowest point in the calibration curve was defined as the lower limit of quantification (LLOQ), which was determined to be at an acceptable accuracy and precision. The recovery of the extractions was evaluated by comparing the areas under the peak of the extracted samples with a non-extracted standard solution. Also, the matrix effect was evaluated by comparing the plasma sample spiked with the analyte with corresponding standard solutions at the same concentrations. Finally, the stability of the sample was investigated at three concentration levels, applying the same chromatographic conditions. The stability tests included long-term, short-term, and freeze-thaw stability.

### Dosing of SER, SER-MP, and collection of the samples

Wistar rats weighing 250 ± 30 g were included in the study. Before oral dosing, the animals were fasted for 12 h. For the dosing solution, 56 mg SER HCl (equivalent to 50 mg SER) was suspended homogenously in 100 mL water containing 0.1% dimethyl sulfoxide (DMSO) to obtain a final concentration of 0.5 mg mL^−1^ of SER (SOL-1). Meanwhile, an equimolar solution of SER-MP was prepared in 0.1% aqueous DMSO to achieve a concentration of 0.8 mg mL^−1^ (SOL-2). The first group of rats received an oral dose of 10.0 mg kg^−1^ (5 mL of SOL-1 per rat weighing 250 ± 30 g) of sertraline. The second group received 16.0 mg kg^−1^ (5 mL of SOL-2 per rat weighing 250 ± 30 g) of SER-MP, equimolar to the SER dose. Blood samples were collected from the ocular vein at 0, 0.5, 1.0, 1.5, 2.0, 3.0, 6.0, 12.0, 24.0, 48.0, 72.0, 120.0, and 190.0 h post-oral dosing and collected into polyethylene tubes containing EDTA-Na. After immediate centrifugation of the blood samples for 15 min at 3500 rpm, the upper clear plasma layer was carefully aspirated using a Pasteur pipette, transferred into an Eppendorf tube, and stored at −80 °C until their analysis. Twenty-four rats were used for the brain sample collection, with samples collected at 1, 2, 6, 24, 48, 72, 120, and 190 h after oral dosing. Phosphate-buffered saline (PBS) was utilized as a matrix to homogenize the brain samples. Both the brain and blood samples were frozen at −80 °C until their analysis.

Wistar rats were obtained from the animal house at the Faculty of Pharmacy, King Abdulaziz University. The Research Ethics Committee, Faculty of Pharmacy, King Abdulaziz University, approved the animal study protocol. The committee ensures all animal use complies with the EU Directive 2010/63/EU on protecting animals used for scientific purposes and the Guiding Principle in Care and Use of Animals (DHEW publication NIH 80-23).^24^

### Analysis of serotonin in brain homogenate

An enzyme-linked immunosorbent assay (ELISA) sandwich kit was used in this study as it is known that it can accurately quantify rat serotonin (ST) in serum, plasma, cell culture supernatants, ascites, tissue homogenates, and other biological fluids. In the assay, first the plate was pre-coated with a rat ST antibody. When ST is present in the sample, it binds to the antibodies on the wells. Next, a biotinylated rat ST antibody was added, which binds to the ST in the sample. Streptavidin-HRP was then added, binding to the biotinylated ST antibody. After incubation, the unbound Streptavidin-HRP was washed away. A substrate solution was added, and it was observed that the color developed proportionally to the amount of rat ST. The reaction was terminated by adding an acidic stop solution, and then the absorbance was measured at 450 nm.

### Statistical analysis of the pharmacokinetic parameters

A noncompartmental model was utilized to analyze the pharmacokinetic parameters of SER. The results were expressed as the mean ± SD. The evaluated parameters included *C*_max_ (maximum concentration), *T*_max_ (time to reach *C*_max_), *t*_1/2_ (half-life), *K*_e_ (elimination rate constant), AUC (area under the concentration–time curve), AUMC (area under the first moment curve), and MRT (mean residence time). The data were analyzed using Kinetica® software, employing a noncompartmental analysis approach. Repeated measure two-way ANOVA was conducted, followed by Sidak's post hoc test, to compare the drug plasma concentration–time curves between the two groups using GraphPad Prism. The pharmacokinetic parameters between the groups were compared using an unpaired two-tailed *t*-test. Statistical significance was defined as *P* < 0.05.

## Result and discussion

### Preparation of SER-MP

The successful synthesis and characterization of the mutual prodrug SER-MP were achieved, marking a significant advancement in developing novel SER formulations. The high yield (90%) of SER-MP demonstrated the efficiency and effectiveness of the synthetic process, providing a solid foundation for further pharmacokinetic investigations.

**Fig. 1:**
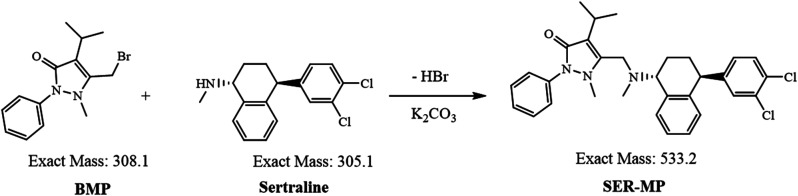
Reaction pathway of SER with BMP.

The reaction likely proceeded *via* nucleophilic substitution of the bromomethyl group in BMP by the amine group in SER, forming SER-MP (Fig. 1). This reaction proceeds *via* the formation of a C–N bond, linking SER to the propyphenazone moiety. The anhydrous potassium carbonate acts as a base and helps deprotonate the amine, making it a better nucleophile and an HBr acceptor. Acetonitrile and chloroform are both suitable solvents for accelerating the reaction. Acetonitrile is polar aprotic, which can stabilize ions, while chloroform is non-polar, which helps to extract organic products. The LC-MS analysis provided evidence for the completeness of the reaction, which was supported by a full scan of the MS^*n*^ spectrum of the reaction product. The theoretical values of log *P*, where *P* expresses the partition coefficient and p*K*a expresses the log of the ionization constant, were calculated theoretically using ChemDraw software (ChemDraw Professional, PerkinElmer informatics Inc., version 16.0.0.82(68), 2016), and were determined to be 5.0 and 9.6 for SER and 6.9 and 7.0 for SER-MP, respectively. The relatively elevated log *P* value of SER-MP suggests its enhanced capacity for penetrating the blood–brain barrier, comparable to that of SER.

The chemical structure was confirmed by MS analysis using an LTQ-XL orbitrap (high-resolution) system; whereby the delta mass was equal to “+*m*/*z* 534.20789 (theoretical) − 534.20038 (found)” = *m*/*z* 0.00751 (Fig. S1†).

Moreover, the chemical structure was confirmed by ^1^H-NMR and ^13^C-NMR in CDCl_3_ using nuclear magnetic resonance (Ultra Shield 600, Bruker, Billerica, MA, USA).

^1^H NMR (600 MHz, CDCl_3_) *δ* 7.81 (d, *J* = 7.8 Hz, 1H), 7.49–7.43 (m, 4H), 7.38–7.32 (m, 2H), 7.27–7.24 (m, 1H), 7.22 (t, *J* = 7.2 Hz, 1H), 7.13 (d, *J* = 6.8 Hz, 1H), 6.95 (d, *J* = 7.2 Hz, 1H), 6.87 (dd, *J* = 6.6, 8.4 Hz, 1H), 4.18 (t, *J* = 4.2 Hz, 1H), 4.09–4.01 (m, 1H), 3.82 (d, *J* = 14.4 Hz, 1H), 3.65 (d, *J* = 13.8 Hz, 1H), 3.24 (s, 3H), 2.90 (t, *J* = 7.2 Hz, 1H), 2.27 (s, 3H), 2.32–2.31 (m, 1H), 2.30–2.22 (m, 1H), 1.86–1.73 (m, 2H), 1.35 (d, *J* = 6.6 Hz, 3H), 1.32 (d, *J* = 7.2 Hz, 3H) (Fig. S2†).

^13^C NMR (150 MHz, CDCl_3_) *δ* 165.39, 152.18, 147.44, 138.82, 138.50, 135.52, 129.19, 128.37, 128.32, 127.40, 126.21, 123.70, 118.22, 60.25, 48.32, 43.76, 37.01, 36.36, 30.18, 29.92, 24.75, 21.44, 21.41, 15.46 (Fig. S3†).

### Analytical method development and validation

We developed and validated a sensitive LC-MS/MS method and an optimized solid-phase extraction procedure that enabled the accurate quantification of SER, SER-MP, 3-OHMP, and the major metabolites in rat plasma and brain homogenate. Various analytical method parameters were evaluated according to international criteria,^23^ including the selectivity, sensitivity, linearity, extraction efficiency, stability, accuracy, and precision. This analytical approach proved crucial for elucidating the prodrug's pharmacokinetic profile and metabolic fate compared to the parent compound SER.

The extraction efficiency was investigated using multiple organic solvents for solid-phase extraction (SPE) to achieve the highest recoveries of SER, SER-MP, and the internal standard (IS) from rat plasma. The optimal extraction efficiency was attained by combining 2 mL ethyl acetate and *n*-hexane (in a 2 : 1 v/v ratio) with 1 mL methanol. This solvent combination facilitated superior blood and brain sample purification during the elution phase.

**Fig. 2:**
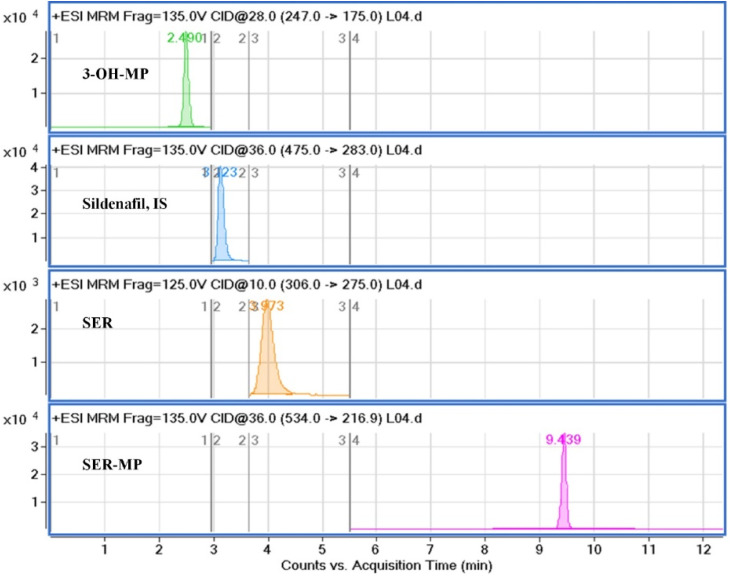
Representative MRM chromatograms of standard sertraline solution, 25 ng mL^−1^ at 3.97 min; SER-MP, 0.25 ng mL^−1^ at 9.43 min; 3-OHMP, 25 ng mL^−1^ at 2.49 min; and internal standard, 20 ng mL^−1^ at 3.22 min.

The mobile phase composition was optimized using a gradient elution method to achieve effective chromatographic separation and analysis. Methanol (solvent A) was found to be superior to acetonitrile and showed better peak symmetry with acceptable retention times. An Eclipse C18 (4.6 mm × 100 mm, 3.5 μm) column enabled excellent chromatographic separation compared with an Eclipse C18 (4.6 mm × 100 mm, 5.0 μm) column. Additional parameters, including the flow rate (0.5 mL min^−1^) and column temperature, were correspondingly optimized. Standard solution mixtures, including of SER, SER-MP, 3-OHMP, and the internal standard were analyzed repeatedly by applying different MRM transitions to ascertain the ideal mass spectrometric parameters. Considering the most stable and abundant product ions, the collision energy (CE), dwell time, and fragmentor voltages were optimized. Details on the optimal MRM parameters are shown in Table 1. The MRM transition chromatograms of the standard compounds (SER, SER-MP, 3-OHMP, and IS) are depicted in Fig. 2.

**Table 1:** MS settings and MRM transitions of the targeted analytes^a^

Compound	*t*_R_, min	Transition, *m*/*z*	CE, *V*	Dwell, sec	Fragmentor, *V*
3-OHMP	2.5	247.1 → 175.0	28	200	135
IS (sildenafil citrate)	3.1	475.2 → 283.0	36	200	135
SER	3.9	306.2 → 275.0	10	160	125
*n*-SER	3.3	292.0 → 275.0	10	160	125
*n*-SER-MP	8.5	519.3 → 217.0	36	200	135
SER-MP	9.4	534.3 → 217.0	36	200	135

a *t*_R_, retention time, CE, collision energy.

### Linearity, limit of detection, and limit of quantitation

The peak area *vs.* the concentration of SER, SER-MP, 3-OHMP, and IS showed a good linear relationship at six concentration levels from 25 ng mL^−1^ to 2000 ng mL^−1^ in rat plasma. The correlation coefficients for the calibration curves of SER, SER-MP, and 3-OHMP were 0.998, 0.9972, and 0.9999, respectively. The response factors, as determined by the slopes, were 0.0002, 0.0013, and 0.0011 MS count per picogram, respectively. The limit of detection (LOD) vales were 8.0, 0.8, and 8.0 ng mL^−1^, and the limit of quantification values were 25, 2.5, and 25 ng mL^−1^ for SER, SER-MP, and 3-OHMP, respectively (Table 2).

**Table 2:** Calibration parameters of SER, SER-MP, and 3-OHMP

Analytes	Regression coefficient, *r*^2^	Relative response factor	Intercept	LOD, ng mL^−1^	LOQ, ng mL^−1^
3-OHMP	0.9999	0.0011	0.0095	8.0	25.0
SER	0.998	0.0002	0.0097	8.0	25.0
SER-MP	0.9972	0.0013	0.0001	0.8	2.5

### Specificity and sensitivity

The proposed HPLC method specificity was evaluated by analyzing blank samples and comparing their chromatographic profiles with actual SER, SER-MP, and 3-OHM samples. Acceptable percentage recoveries were achieved for all analytes, with no interfering peaks observed. Excellent separation was achieved between SER, SER-MP, 3-OHMP, and IS, with sharp peaks and high resolution. The retention times were 3.97, 9.43, 2.49, and 3.22 min for SER, SER-MP, 3-OHMP, and IS, respectively. No impurities were detected throughout the retention time window, demonstrating the HPLC method's good ability to selectively determine SER, SER-MP, and 3-OHMP without interference from co-formulation adjuvants.

### Recovery

The recovery percentage was determined at three concentration levels of QC samples by comparing the mean peak area ratios of the recovered analyte from the spiked sample divided by the mean peak area of the neat samples (without extraction) and multiplied by 100%. The percentage recoveries of SER, SER-MP, and 3-OHMP were not less than 90% for all entities, as shown in Table 3. This data revealed our proposed method enabled efficient extraction compared to other reported ones.

**Table 3:** Evaluation of the intraday and interday accuracy, QC sample stability, and SER, SER-MP, and 3-OHMP precision in spiked rat plasma

Concentration in plasma, ng mL^−1^	Average recovery (%) ± SD		Intraday		Interday	
	Autosampler (25 °C/24 h)	Freeze-thaw (−20 °C/3 cycles)	Recovery %^a^ (RSD)	Er^b^ (%)	Recovery %^a^ (RSD)	Er^b^ (%)
**SER**						
50.0	92.98 ± 1.35	91.04 ± 2.95	91.94 (0.53)	−8.06	90.84 (0.84)	−9.16
500.0	93.23 ± 0.98	90.68 ± 3.92	92.42 (0.74)	−7.58	91.36 (0.57)	−8.64
2000.0	91.15 ± 2.20	89.23 ± 4.00	93.44 (0.48)	−6.56	91.56 (0.90)	−8.44

**SER-MP**						
50.0	97.45 ± 0.78	95.70 ± 1.76	98.11 (0.57)	−1.89	97.71 (0.35)	−2.29
500.0	98.76 ± 1.03	96.84 ± 0.68	97.27 (0.67)	−2.27	96.83 (0.56)	−3.17
2000.0	98.88 ± 0.80	94.99 ± 2.09	98.03 (1.08)	−1.60	97.07 (0.36)	−2.93

**3-OHMP**						
50.0	96.80 ± 0.98	94.39 ± 2.98	96.61 (0.42)	−3.39	96.24 (0.59)	−3.76
500.0	96.55 ± 1.20	95.47 ± 1.57	95.97 (0.41)	−4.03	94.75 (0.71)	−5.25
2000.0	95.24 ± 2.56	95.73 ± 1.93	96.40 (0.61)	−3.60	95.95 (0.11)	−4.05

a Mean recovery % (RSD) of three determinations.

b Percentage relative error.

### Precision and accuracy

To ensure the reliability and consistency of the analytical method for SER, SER-MP, and 3-OHMP, both the intraday and interday precision and accuracy were evaluated. This test involved analyzing quality control (QC) samples at three different SER, SER-MP, and 3-OHM concentrations. The results of this evaluation are summarized in Table 3. The intraday precision was assessed by repeating the assay three times in one day, while the interday precision was evaluated by analyzing sets of samples on three different days. The RSD% values displayed in Table 3 indicate the variability of the measurements. The accuracy of the analysis was determined by comparing the measured concentrations of the analytes in the QC samples to their true values. The Er% values in Table 3 indicate how close the measured concentrations were to the expected values. Generally, RSD% values below 15% and Er% values within ±10% are considered acceptable in terms of precision and accuracy, respectively. The results obtained in our study were well within these limits, indicating out method was a reliable and consistent analytical method for SER, SER-MP, and 3-OHMP analysis.

### Stability study

The stability of the standard solutions containing SER and SER-MP was assessed under different storage and temperature conditions. Standard solutions were maintained at room temperature for 6 h and 4 °C for 12 h, and then analyzed using the developed method. No significant changes in peak area values were observed throughout the analysis, indicating the excellent stability at both temperatures. Additionally, samples subjected to three freeze-thaw cycles (−20 °C) showed no significant degradation. Recoveries remained above 89%, and the standard deviation (SD%) values were below 3%, as detailed in Table 3.

### Matrix effect

Next, we examined how the co-extracted biogenic components affected the analytes' ionization efficiency in the ESI compartment. For this, a plasma sample containing 10 ng mL^−1^ of each analyte was prepared and extracted. Meanwhile, an equivalent standard solution was prepared in methanol. The sample and standard solution were analyzed in the +LC-MS MRM mode to match the ESI-MS response. The calculated average response of all analytes was in the range from 98.3–99.2 ± 0.63%.

### Pharmacokinetic parameters

**Fig. 3:**
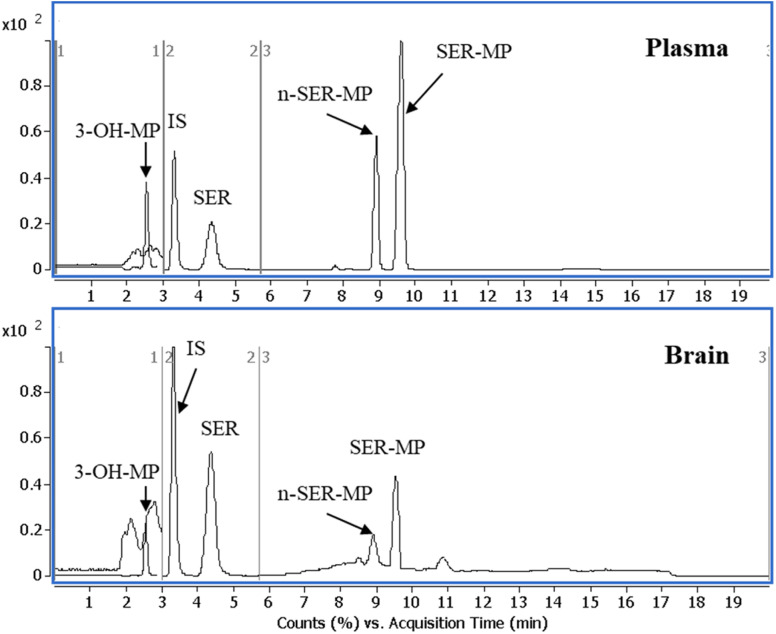
Representative base peak MRM overlay-chromatogram of extracted rat plasma and brain after the oral administration of SER-MP.

Fig. 3 displays a representative base peak MRM overlay-chromatogram for SER, SER-MP, and their major metabolites extracted from rat plasma and brain after medication. The concentration of the *N*-desmethyl metabolite of SER-MP (*n*-SER-MP, *m*/*z* 520) was calculated from the calibration curve of SER-MP (Fig. 3).

**Fig. 4:**
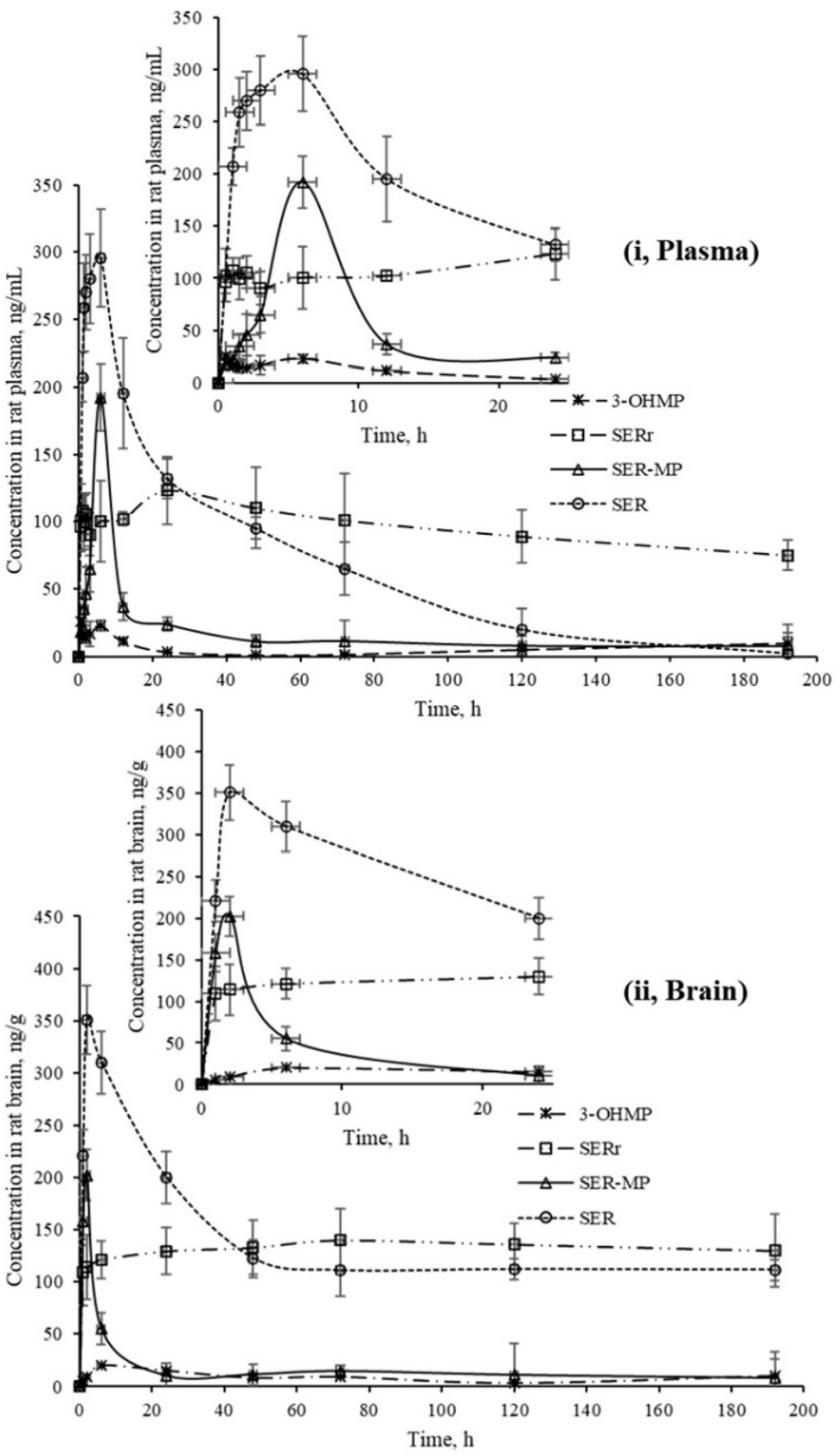
Concentrations of SER, SER-MP and its metabolites in rat plasma (i), and brain homogenate (ii).

Fig. 4 shows that SER's plasma and brain concentration profile differed from that of the SER released from SER-MP. Specifically, the concentration level of the released SER in plasma was higher and more steady than that of the orally administered SER. These results support the hypothesis that the prepared prodrug saves SER from the intensive first-path effect. A separate figure (Fig. 4) shows that the nor-methyl-SER-MP was the major metabolite of SER-MP, with a higher concentration level in the plasma, and that it could also be retained in the plasma for a long time, reaching 195 h.

The calculated concentrations were used to estimate the pharmacokinetic parameters of the orally administered SER and SER-MP in rats. Here, the plasma (i) curves of SER showed it underwent a rapid absorption and elimination compared with SER-MP, demonstrating a more sustained plasma concentration over time. Meanwhile, the brain homogenate (ii) curve showed that SER-MP maintained a more consistent brain concentration than SER. These results suggest the improved brain penetration and retention of SER-MP. The pharmacokinetic parameters (noncompartmental model) calculated from the rat plasma data are summarized in Table 4. It could be seen that SER and SER-r displayed similar elimination rates (*k* = 0.3), while SER-MP had a slower rate (*k* = 0.1).

**Table 4:** Rat plasma pharmacokinetic parameters of the investigated compounds after their oral administration^a^

Parameter	Unit	Value ± SD, of orally administered		
		SER	SER-r*	SER-MP
*K*_e_	h^−1^	0.03	0.03	0.01
*t*_1/2_	h	23.61	27.05	49.97
*T*_max_	h	6.00	24.00	6.00
*C*_max_	ng mL^−1^	295.55	123.02	192.11
AUC_0−*t*_	ng mL^−1^ h	13 204.20	10 625.66	3092.63
AUC_0-inf_obs_	ng mL^−1^ h	13 289.35	10 723.24	3251.22
MRT_0-inf_obs_	h	45.36	51.08	55.23
*C*_max_/AUC_(0–24)_	μg h L^−1^	0.03	0.04	0.22
Total clearance rate	mL min^−1^ kg^−1^	0.00075	0.00093	0.0030

a Abbreviations: *K*_e_; elimination rate constant, *t*_1/2_ half-life, *T*_max_; time to reach maximum plasma concentration, *C*_max_; maximum plasma concentration, AUC, the area under the plasma concentration–time curve, MRT, mean residence time, SER-r*: sertraline released from SER-MP as a metabolite.

**Fig. 5:**
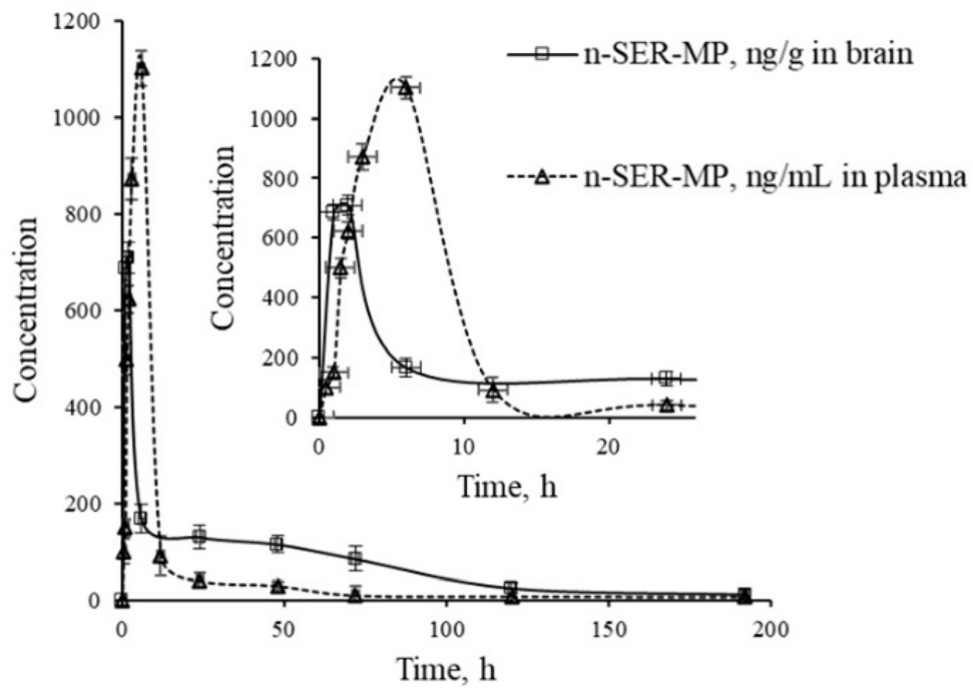
Levels of *n*-SER-MP metabolite in rat plasma and brain following the oral administration of SER-MP.

SER and SER-MP reached peak plasma concentrations (*C*_max_) of 295 and 192 ng mL^−1^ at 6 h post-dosing, whereas 3-OHMP peaked at 123 ng mL^−1^ after 24 h. The elimination half-life of SER-MP exhibited the longest half-life (*t*_1/2_ = 50 h), followed by SER-r (*t*_1/2_ = 27 h) and SER (*t*_1/2_ = 23 h). The orally administered SER displayed the highest AUC in plasma (13 204 ng mL^−1^ h^−1^), followed by SER-MP (AUC, 3092 ng mL^−1^ h^−1^) and 3-OHMP (AUC, 10 625 ng mL^−1^ h^−1^). The plasma level of intact sertraline released as a metabolite of orally administered SER-MP was increased 2.4-folds compared with the administered SER at an equimolar dose. The concentration of SER, derived from either orally administered SER or its derivative SER-MP, was measured and found to be between 100 and 170 ng g^−1^ in the brain homogenate of the rats. The prepared prodrug existed at a steady range around 100 ng g^−1^ in the brains of the rats from 0.5 to 192 h (Fig. 5).

The profile of the obtained pharmacokinetic data of the propyphenazone-based SER-prodrug supported the hypothesis of the safe and sustained delivery of SER, which could reduce the probable spiky elevation in the concentration of SER in plasma or brain. The propyphenazone moiety may also alter the conjugated drug's pharmacokinetics, potentially extending its duration of action or improving its distribution to target tissues. These mechanisms may allow propyphenazone derivatives to potentially enhance efficacy through targeted effects while mitigating the safety concerns typically associated with SER.

### Serotonin levels in the brain homogenate

**Fig. 6:**
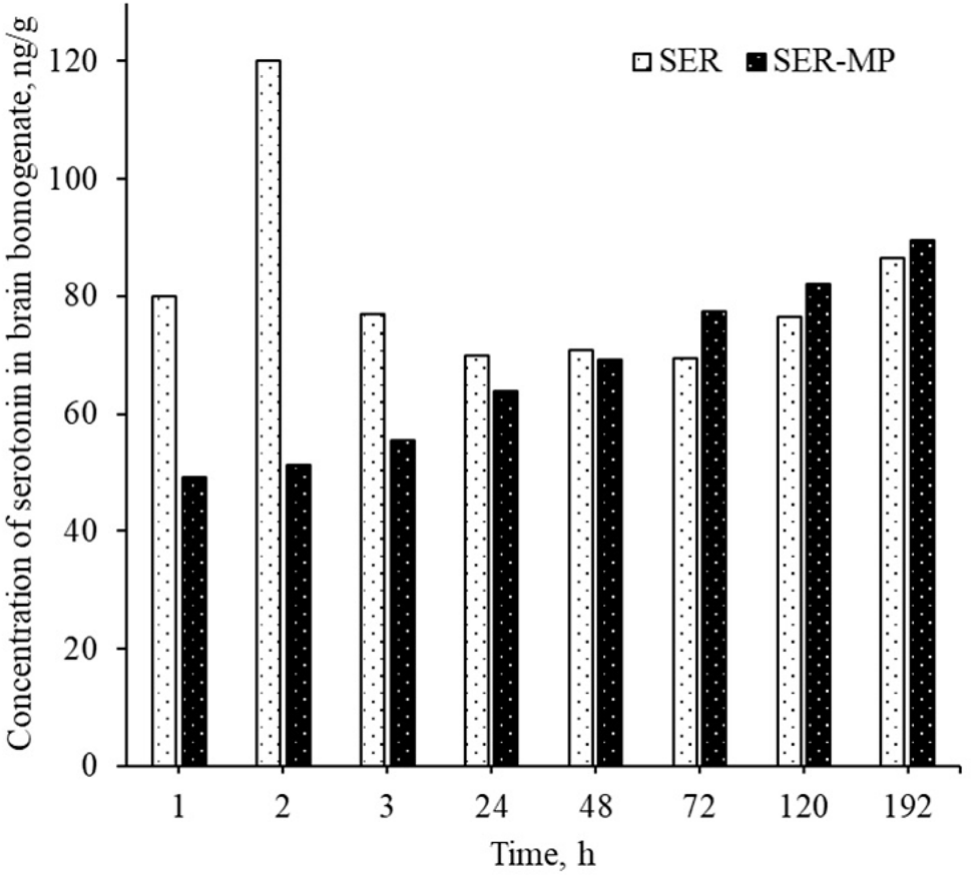
Concentration of serotonin in the rat brain homogenates for a rat receiving an oral equimolar dose of SER and SER-MP.

The serotonin level in rat brain homogenate was within the range of 50–90 ng g^−1^ for both the group receiving SER and the group receiving an equal molar dose of SER-MP, as shown in Fig. 6. This observation was consistent within a time range of 1–192 h from oral administration.

An abnormal increase in the serotonin level in the brain was observed within 1 to 3 h after the oral administration of a single dose of SER. This sudden spike in serotonin levels in the brain can lead to a condition known as serotonin syndrome. This condition can manifest with an autonomic instability that involves a rapid heart rate, high blood pressure, and hyperthermia.^9^ Patients may normally suffer from serotonin syndrome for 1 to 4 weeks until a steady level is attained, depending on the personal variation in metabolism and the medications used by the patients.^10^ However, the rats receiving an oral dose of SER-MP in the present study showed an approximately steady level of serotonin in the brain, as shown in Fig. 6. This finding suggests that utilizing SER-MP rather than SER can help avoid major life-threatening adverse effects caused by a sudden spike in serotonin levels in the brain.

### Confirmation of the extracted metabolites in rat plasma and brain

**Fig. 7:**
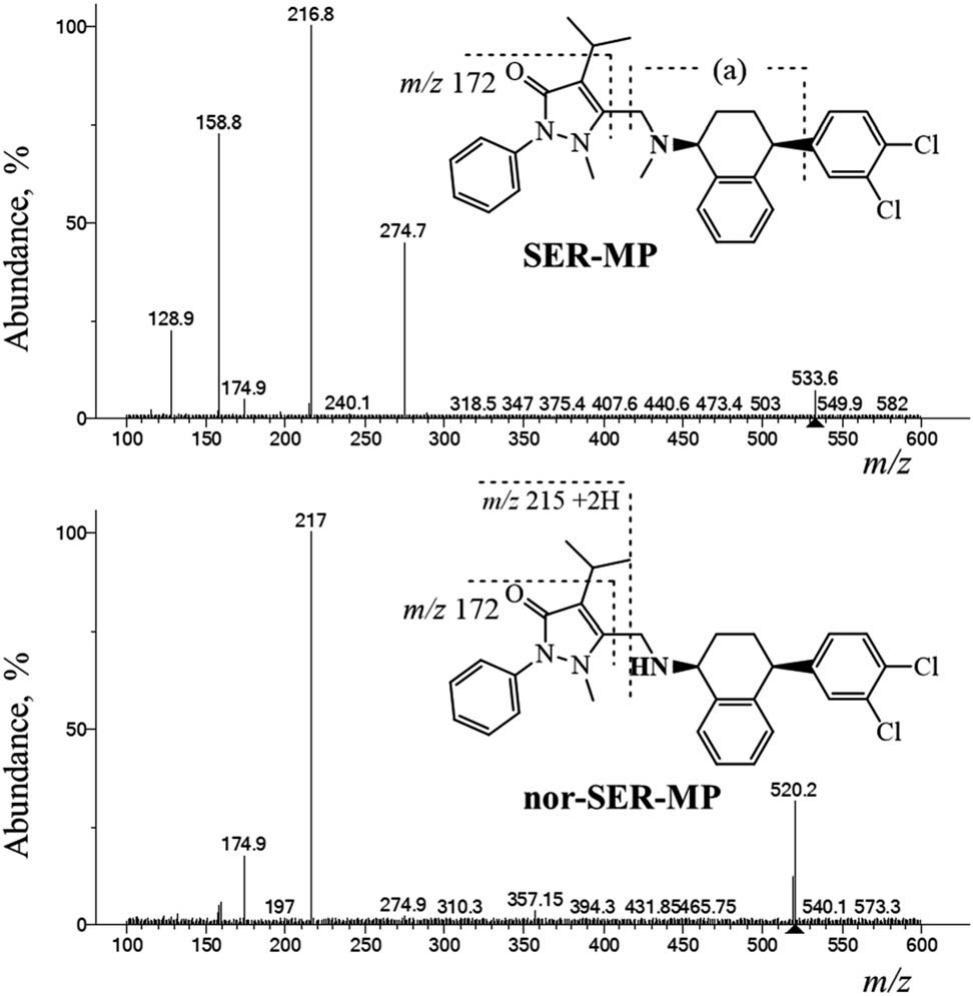
QqQ-MS^2^ spectra and fragmentation pathway of SER-MP and its desmethyl-metabolites.

The SER-MP metabolites were analyzed using positive electrospray ionization mass spectrometry (ESI-MS) followed by fragmentation at a collision energy of 36 eV using liquid chromatography-triple quadrupole mass spectrometry (LC-QqQ-MS) over a mass range of 100–600 *m*/*z*. The LC-MS data analysis revealed distinct chromatographic peaks corresponding to the primary metabolites. NIST-Interpreter software was used to interpret the resulting MS^2^ spectra. The metabolites, confirmed through the fragmentation pathway illustrated in Fig. 7, facilitated the interpretation and verification of SER-MP and its metabolites in the plasma and brain samples, with the major metabolites identified as *N*-desmethyl-SER-MP, 3-OHMP, and SER (assigned as SER released). The MS^2^ spectrum of the metabolite with a mass-to-charge ratio (+*m*/*z*) of 520 showed base-peak fragment ions at *m*/*z* 217. This comprehensive analysis allowed determining the metabolite concentrations, which ranged from 100–1100 ng mL^−1^ in plasma and 100–700 ng mL^−1^ in brain tissue.

The base peak of *m*/*z* 217 corresponded to the propyphenazone moiety +2H. However, the peak at *m*/*z* 159 corresponded to [fragment (a) “*m*/*z* 173” − CH_3_ + H] and reached 74% abundance in the MS^2^ spectrum of SER-MP. *n*-SER-MP showed <10% abundance of the same peak at *m*/*z* 159, which corresponded to the [propyphenazone “*m*/*z* 215” − isopropyl “*m*/*z* 43” − CH_3_ + 2H]. The high % abundance of *m*/*z* 159 in the SER-MP spectrum compared to in the spectrum for *n*-SER-MP confirmed the absence of the methyl group attached to the peripheral amino group in the SER moiety.

## Conclusion

A prodrug of SER was synthesized by conjugating it with MP *via* the amino group to enhance its lipophilicity and first-pass stability. A validated liquid chromatography-tandem mass spectrometry (LC-MS/MS) method was developed to profile the metabolic fate of the administered SER *versus* SER-MP. Additionally, a solid-phase extraction (SPE) method was optimized to achieve acceptable recovery with minimal sample contamination. The pharmacokinetic evaluation of SER-MP compared to sertraline (SER) in rat models revealed several promising outcomes, including an enhanced plasma concentration, prolonged half-life, sustained brain concentration, and steady serotonin levels. The plasma level of SER released from SER-MP was 2.4 times higher than that from orally administered SER at equimolar doses. SER-MP also exhibited a longer elimination half-life (50 h) than SER (23 h) and could also maintain a stable concentration of approximately 100 ng g^−1^ in rat brains from 0.5 to 192 h post-administration. The serotonin levels in rat brain homogenates remained consistent (50–90 ng g^−1^) for both the SER and SER-MP groups over 192 h. These findings suggest that the SER-MP prodrug could offer several advantages over traditional sertraline formulations, including improved pharmacokinetics, potentially reduced side effects, and possibly enhanced therapeutic efficacy. However, further research is necessary to fully elucidate the long-term effects, safety profile, and clinical efficacy of SER-MP in human subjects. Overall, the SER-MP prodrug shows promise as an innovative approach for enhancing the pharmacokinetic properties and potentially the therapeutic index of sertraline, paving the way for more effective and safer treatments for depression and related disorders.

## Data availability

Data are available on request.

## Conflicts of interest

There are no conflicts to declare.

## Supplementary Material

RA-015-D4RA08357A-s001
