# Supplementary material for: Preparation and pharmacokinetic evaluation of a sertraline-methylpropyphenazone prodrug: a comparative metabolic study on the plasma and brain tissues of rats using LC-MS/MS analysis
Source: RSC Adv. 2025 Jan 28;15(4):2800–9. doi: 10.1039/d4ra08357a (PMC11773334; doi:10.1039/d4ra08357a)
Supplement: RA-015-D4RA08357A-s001 [file RA-015-D4RA08357A-s001.pdf]

Supplementary Information (SI) for RSC Advances.
This journal is © The Royal Society of Chemistry 2025

Supplementary data

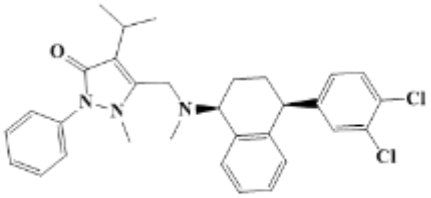

Sertraline – methyl-propyphenazone (SER-MP)

Purification

The compound was purified by column chromatography over silica gel, mobile phase dichloromethane (DCM), and then 9:1 DCM/ethyl acetate. The obtained purity, as an oily substance, reached 98.8%, as determined by HPLC-DAD at 254 nm.

High-Resolution Mass of SER-MP

Instrument

Thermo Scientific LTQ-XL Orbitrap mass spectrometer coupled with Accela autosampler and Accela pump (San Jose, CA, USA). The ion source; electrospray ionization (ESI) compartment. The system was controlled with Xcalibur® Thermo Fisher Scientific Inc, version 2.07 SP1. Spray voltage, 5.0 kv, sheath gas flow rate, 42 mL/min, auxiliary gas, 5 mL/min, capillary voltage, 60v, capillary temperature, 335 °C, scan range, 100 - 700 m/z. The collision energy was 35 v. Column, Eclipse Plus C18, 3.5 µm, 4.6X100 mm (Agilent, Palo Alto, USA), column oven, 25 °C. The mobile system comprised acetonitrile (90%), water containing 0.1% glacial acetic acid 100% (10%). The flow rate was 400 µL/min. A positive ESI-MS mode was applied.

Result

The delta mass = +*m/z* 534.20789- 534.20038 = *m/z* 0.00751

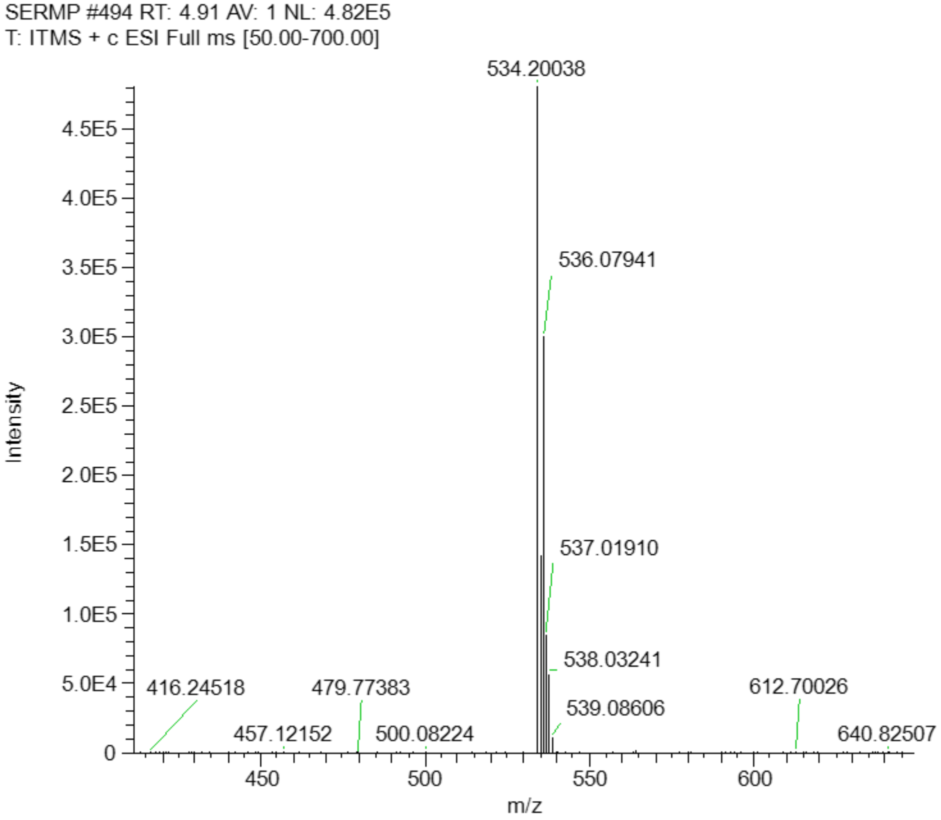

Fig. S1. Mass spectrum of SER-MP

^{1}H-NMR & ^{13}C-NMR analysis

The 1H-NMR and 13C-NMR analysis was repeated using Nuclear Magnetic Resonance, Ultra Shield 600, Bruker, Billerica, Massachusetts, United States.
The 600 MHz NMR data was added (Fig. S2 & Fig. S3).
Adjusted paragraph:
^{1}H NMR (600 MHz, CDCl3) δ 7.81 (d, J = 7.8 Hz, 1H), 7.49 – 7.43 (m, 4H), 7.38 – 7.32 (m, 2H), 7.27 – 7.24 (m, 1H), 7.22 (t, J = 7.2 Hz, 1H), 7.13 (d, J = 6.8 Hz, 1H), 6.95 (d, J = 7.2 Hz, 1H), 6.87 (dd, J = 6.6, 8.4 Hz, 1H), 4.18 (t, J = 4.2 Hz, 1H), 4.09 – 4.01 (m, 1H), 3.82 (d, J = 14.4 Hz, 1H), 3.65 (d, J = 13.8 Hz, 1H), 3.24 (s, 3H), 2.90 (t, J = 7.2 Hz, 1H), 2.27 (s, 3H), 2.32 – 2.31 (m, 1H), 2.30 – 2.22 (m, 1H), 1.86 – 1.73 (m, 2H), 1.35 (d, J = 6.6 Hz, 3H), 1.32 (d, J = 7.2 Hz, 3H) (Fig. S2).

^{13}C NMR (150 MHz, CDCl3) δ 165.39, 152.18, 147.44, 138.82, 138.50, 135.52, 129.19, 128.37, 128.32, 127.40, 126.21, 123.70, 118.22, 60.25, 48.32, 43.76, 37.01, 36.36, 30.18, 29.92, 24.75, 21.44, 21.41, 15.46 (Fig. S3).

Fig. S2. ^{1}H NMR spectrum of SER-MP in CDCl3

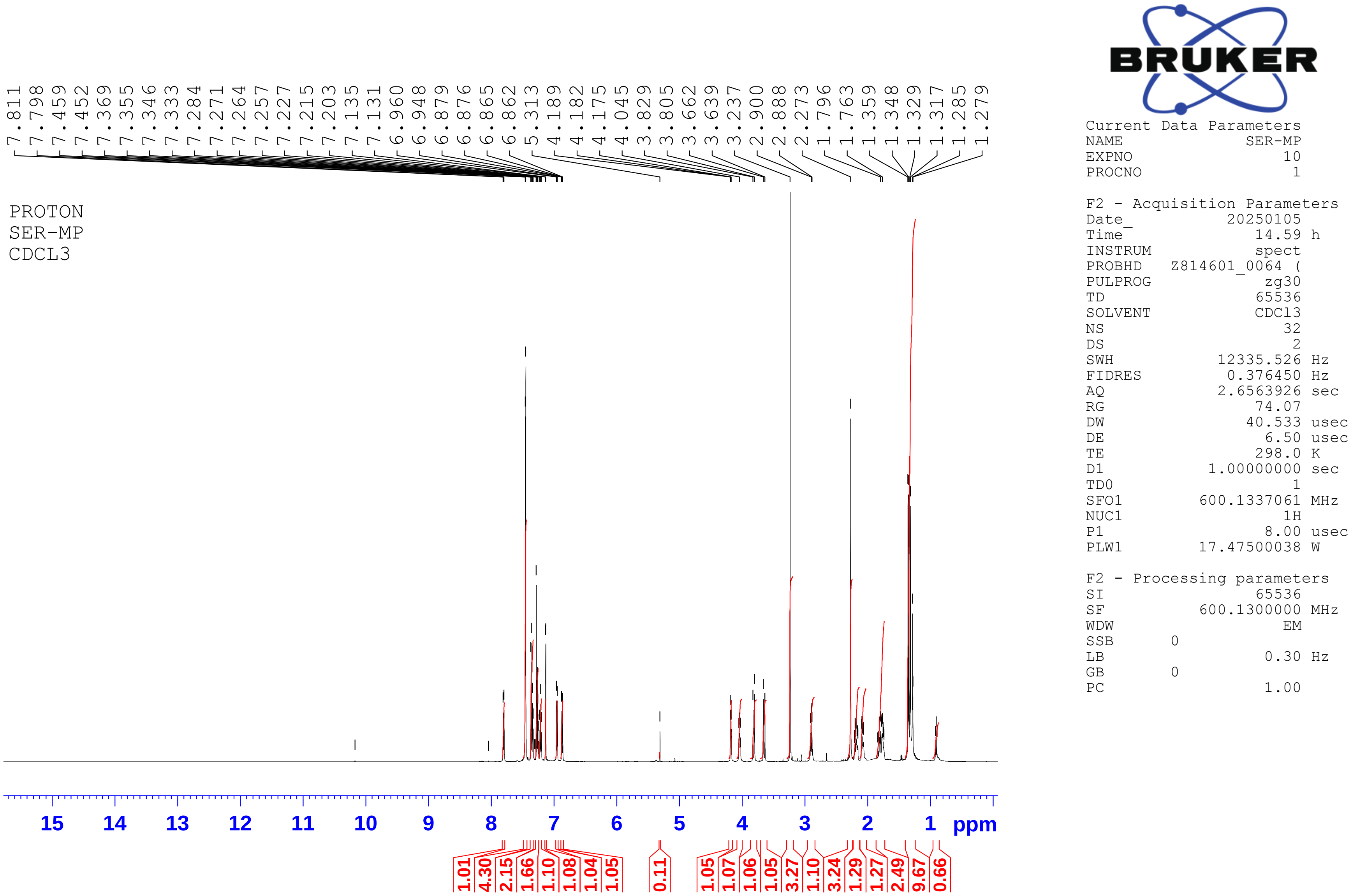

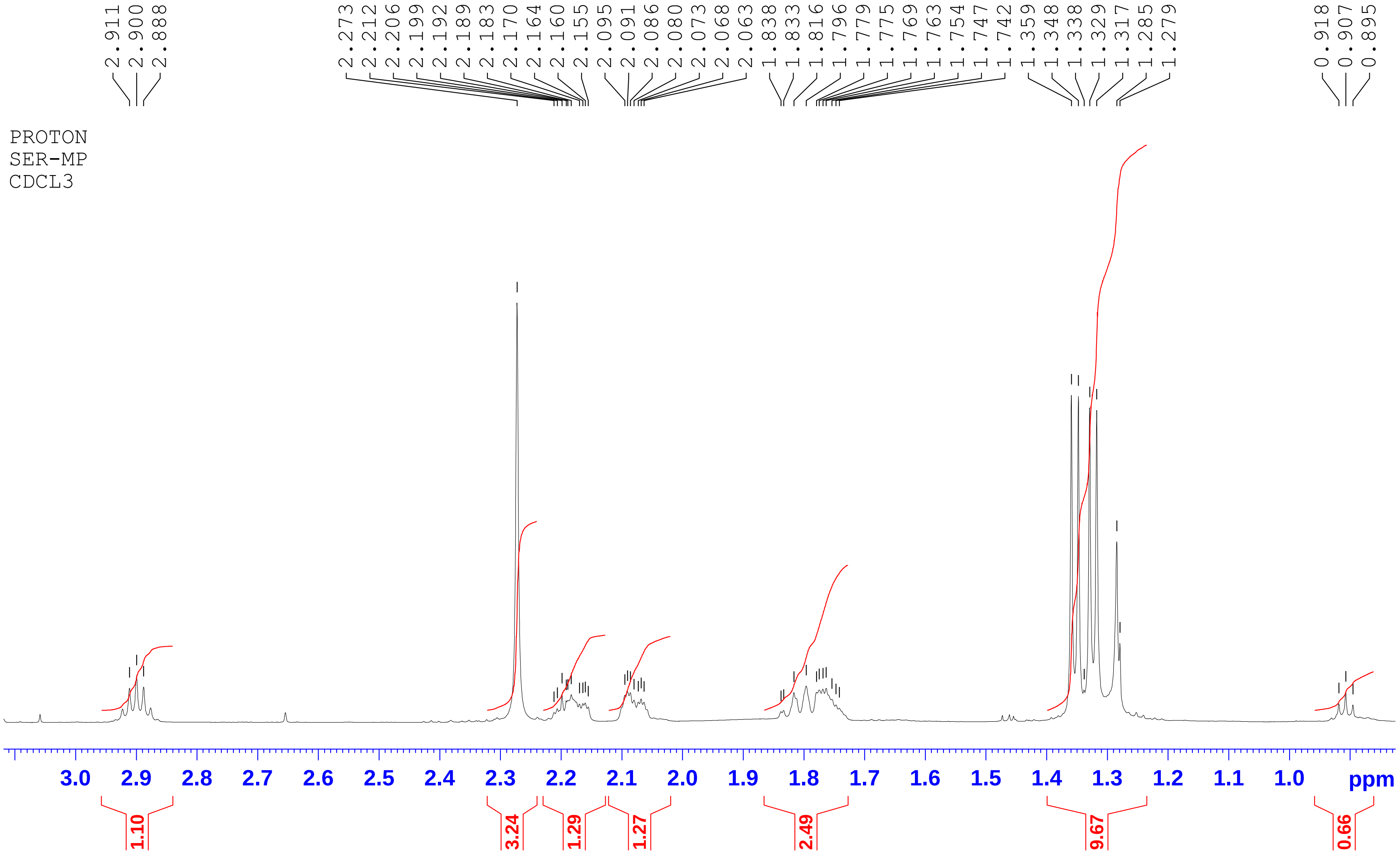
PROTON
SER-MP
CDCL3
2.911 2.900 2.888
2.273 2.212 2.206 2.199 2.192 2.189 2.183 2.170 2.164 2.160 2.155
2.095 2.091 2.086 2.080 2.073 2.068 2.063
1.838 1.833 1.816 1.796 1.779 1.775 1.769 1.763 1.754 1.747 1.742
1.359 1.348 1.338 1.329 1.317 1.285 1.279
0.918 0.907 0.895
3.0 2.9 2.8 2.7 2.6 2.5 2.4 2.3 2.2 2.1 2.0 1.9 1.8 1.7 1.6 1.5 1.4 1.3 1.2 1.1 1.0 ppm
1.10 3.24 1.29 1.27 2.49 9.67 0.66

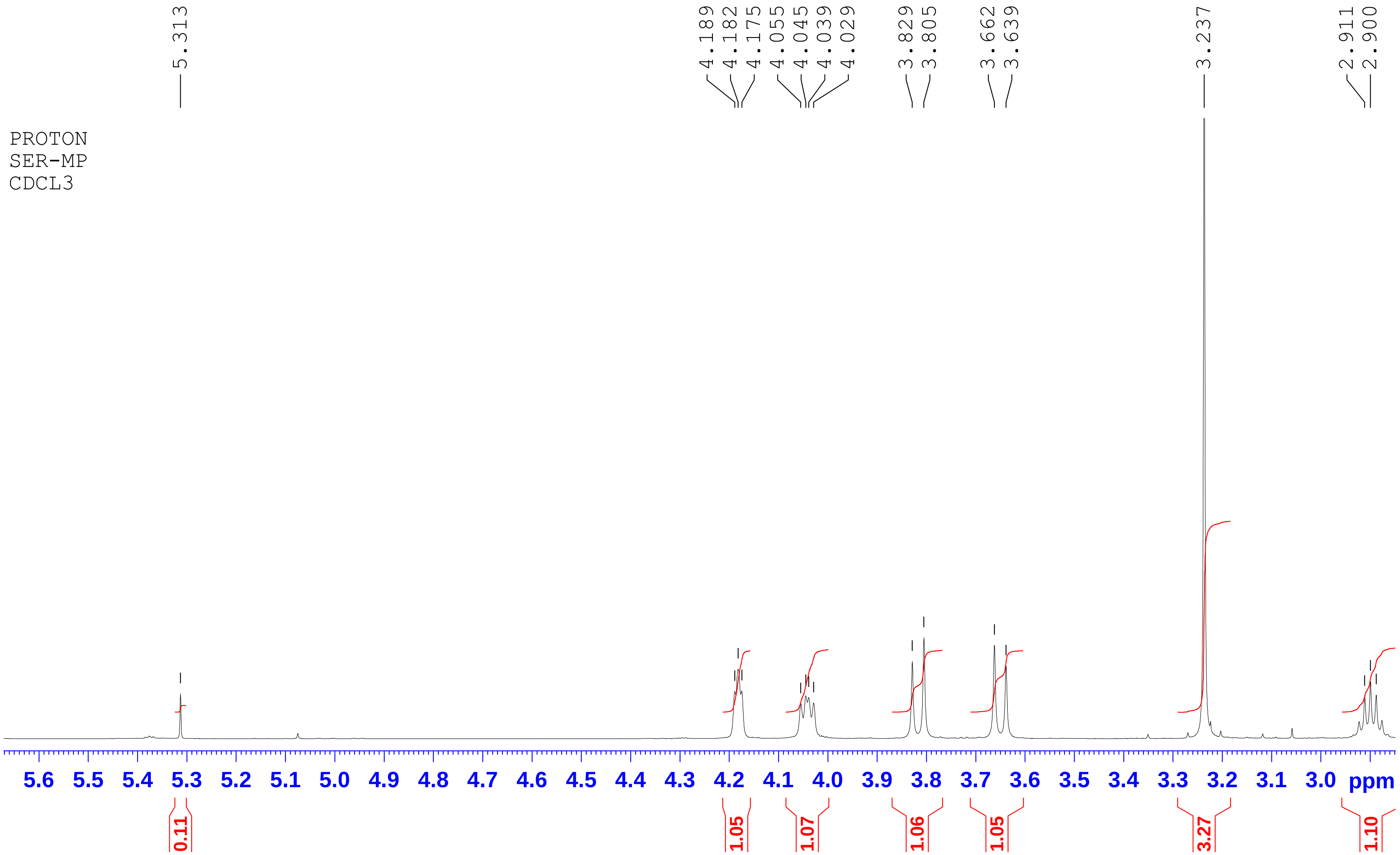
5.313
4.189
4.182
4.175
4.055
4.045
4.039
4.029
3.829
3.805
3.662
3.639
3.237
2.911
2.900
PROTON
SER-MP
CDCL3
5.6 5.5 5.4 5.3 5.2 5.1 5.0 4.9 4.8 4.7 4.6 4.5 4.4 4.3 4.2 4.1 4.0 3.9 3.8 3.7 3.6 3.5 3.4 3.3 3.2 3.1 3.0 ppm
0.11
1.05
1.07
1.06
1.05
3.27
1.10

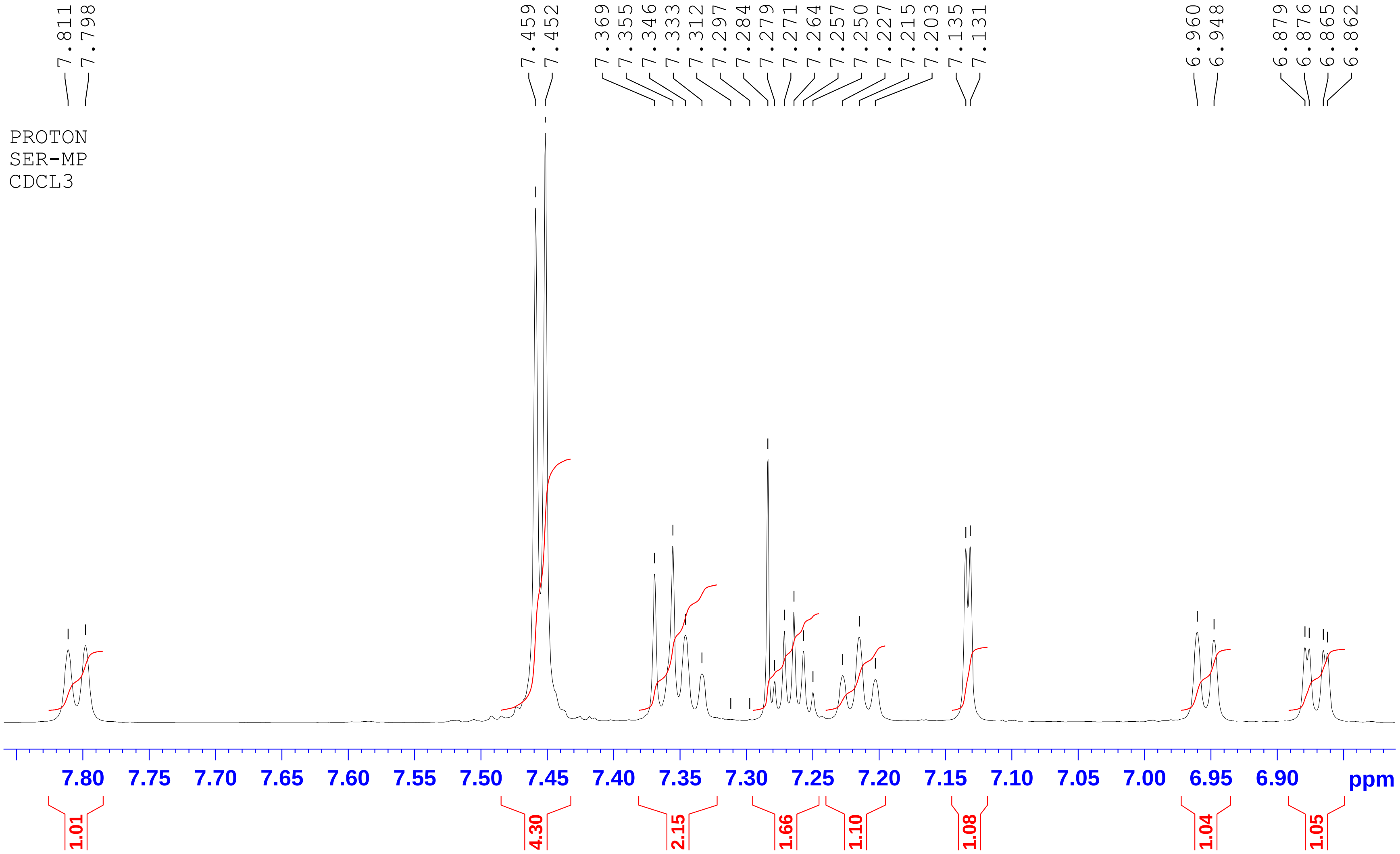
PROTON
SER-MP
CDCL3
7.811
7.798
7.459
7.452
7.369
7.355
7.346
7.333
7.312
7.297
7.284
7.279
7.271
7.264
7.257
7.250
7.227
7.215
7.203
7.135
7.131
6.960
6.948
6.879
6.876
6.865
6.862
7.80
7.75
7.70
7.65
7.60
7.55
7.50
7.45
7.40
7.35
7.30
7.25
7.20
7.15
7.10
7.05
7.00
6.95
6.90
ppm
1.01
4.30
2.15
1.66
1.10
1.08
1.04
1.05

.Fig. S3. ^{13}C NMR spectrum of SER-MP in CDCl3

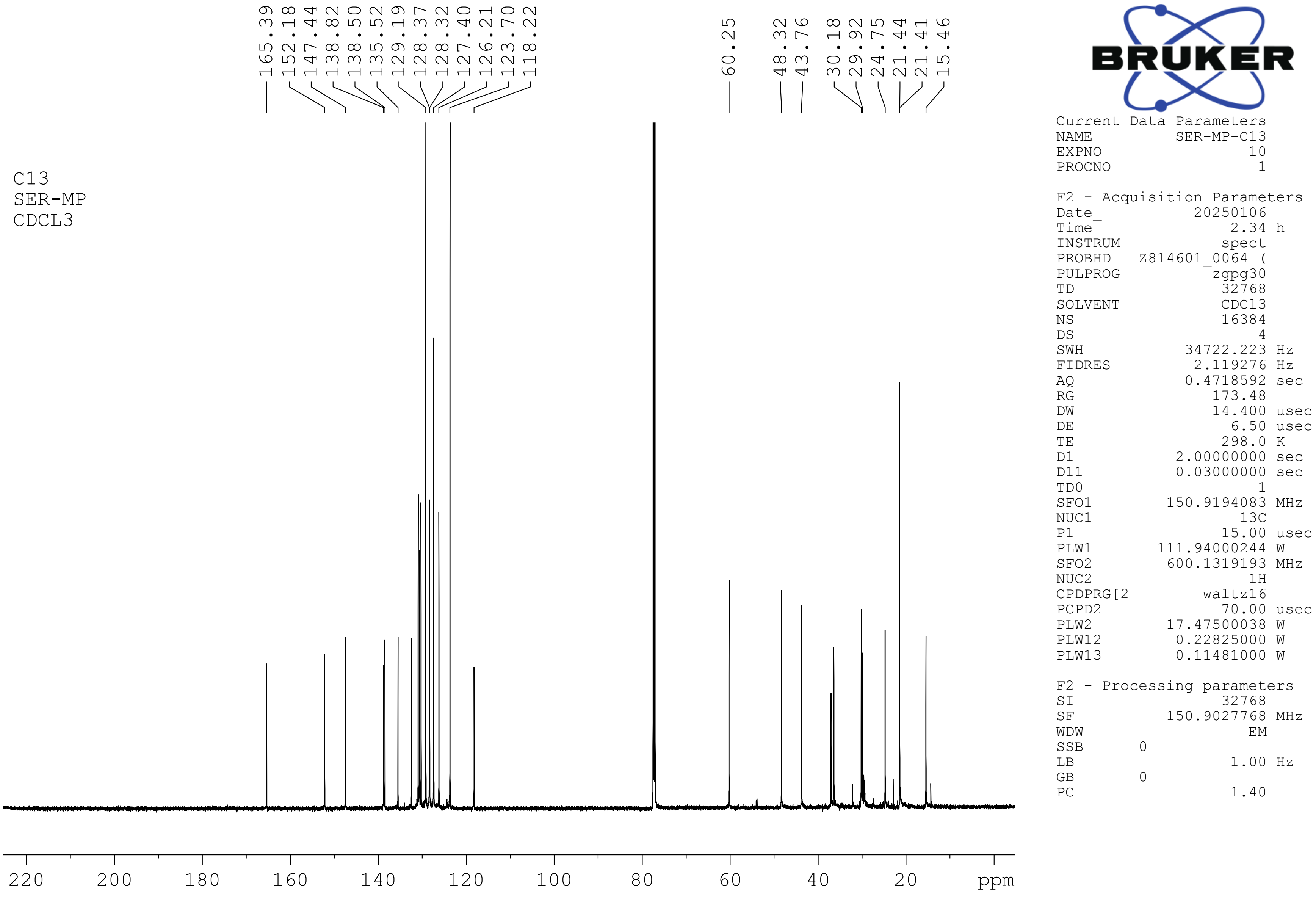

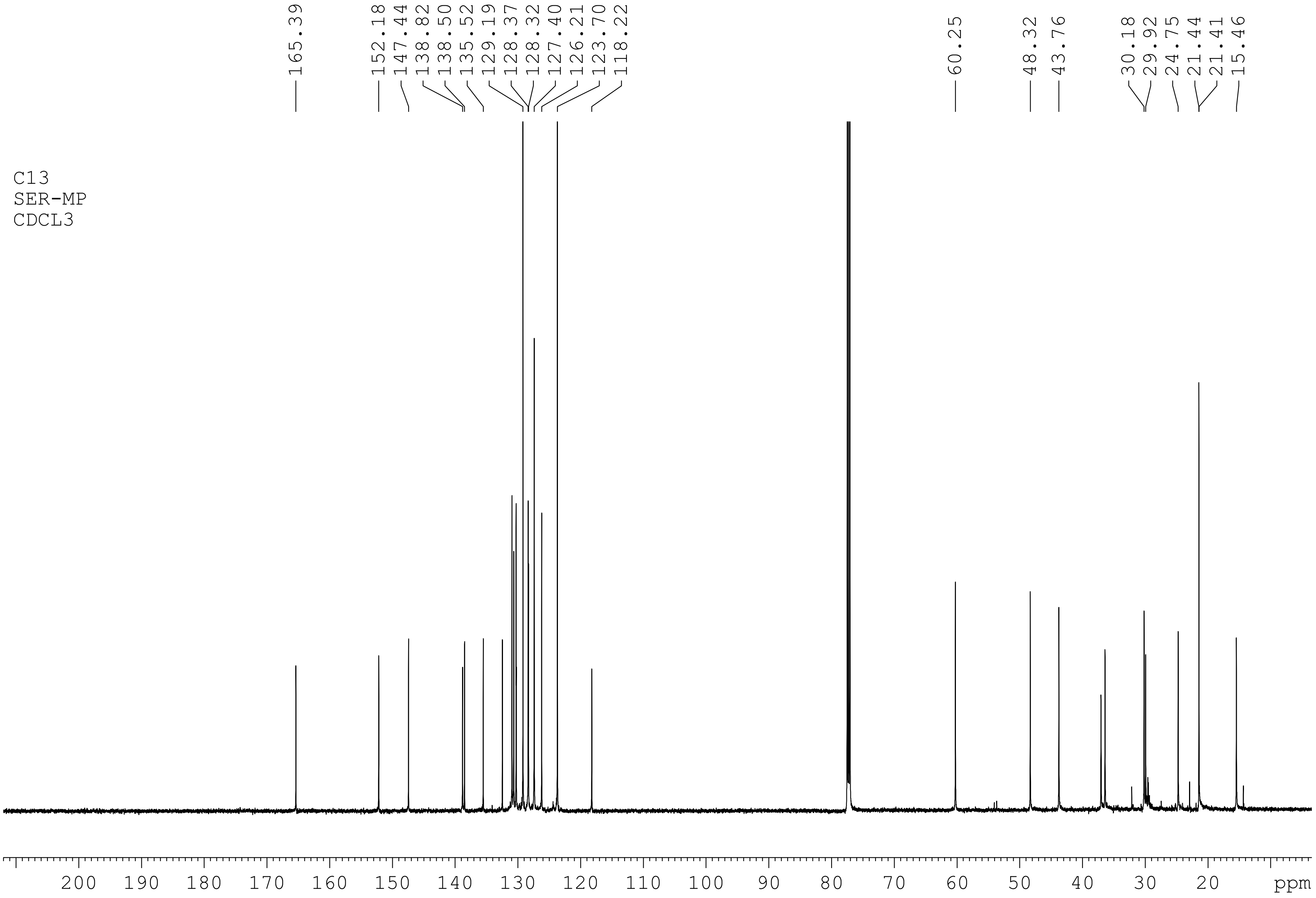

165.39
152.18
147.44
138.82
138.50
135.52
129.19
128.37
128.32
127.40
126.21
123.70
118.22
60.25
48.32
43.76
30.18
29.92
24.75
21.44
21.41
15.46
C13
SER-MP
CDCL3
200 190 180 170 160 150 140 130 120 110 100 90 80 70 60 50 40 30 20 ppm

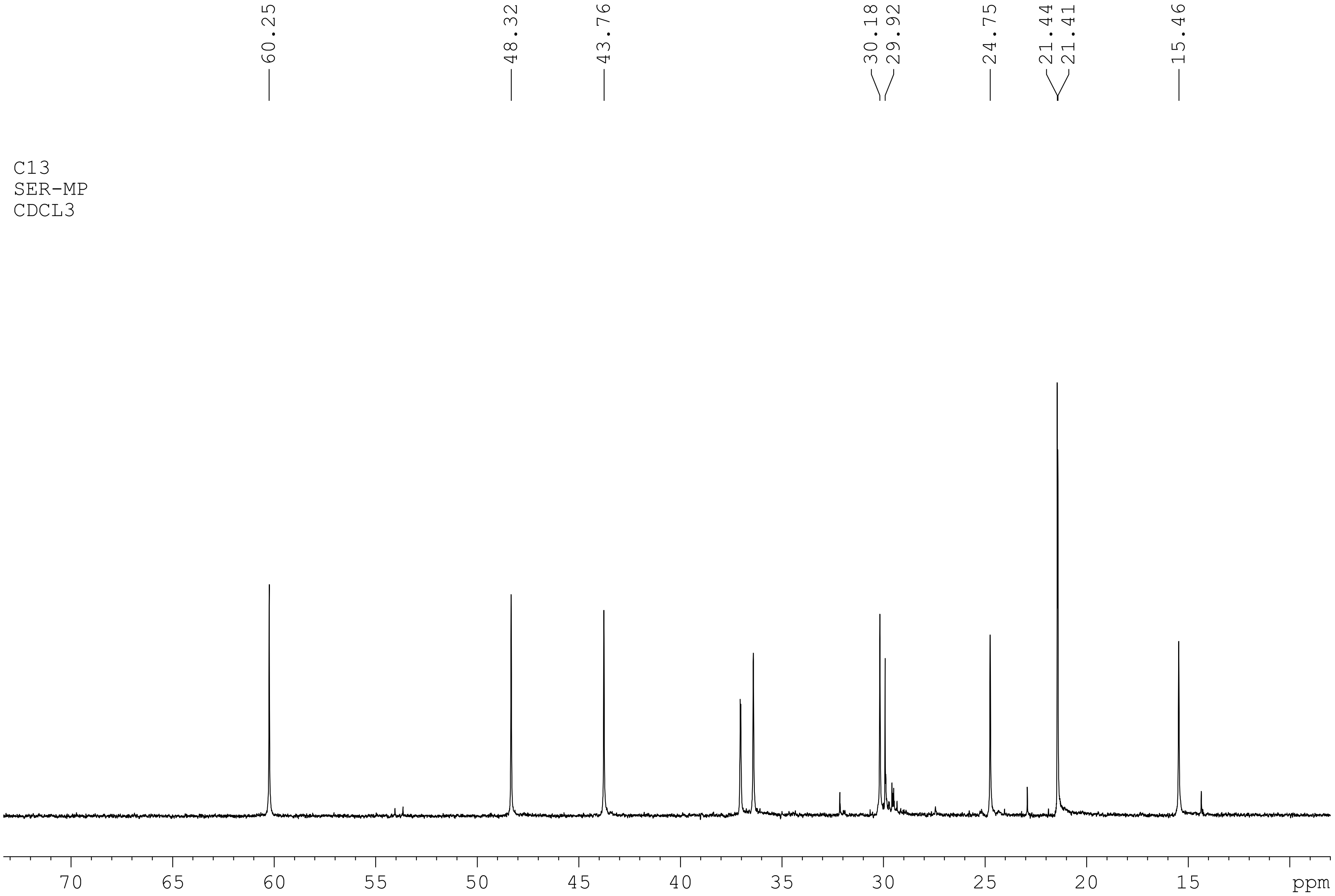
60.25
48.32
43.76
30.18
29.92
24.75
21.44
21.41
15.46
C13
SER-MP
CDCL3
70
65
60
55
50
45
40
35
30
25
20
15
ppm

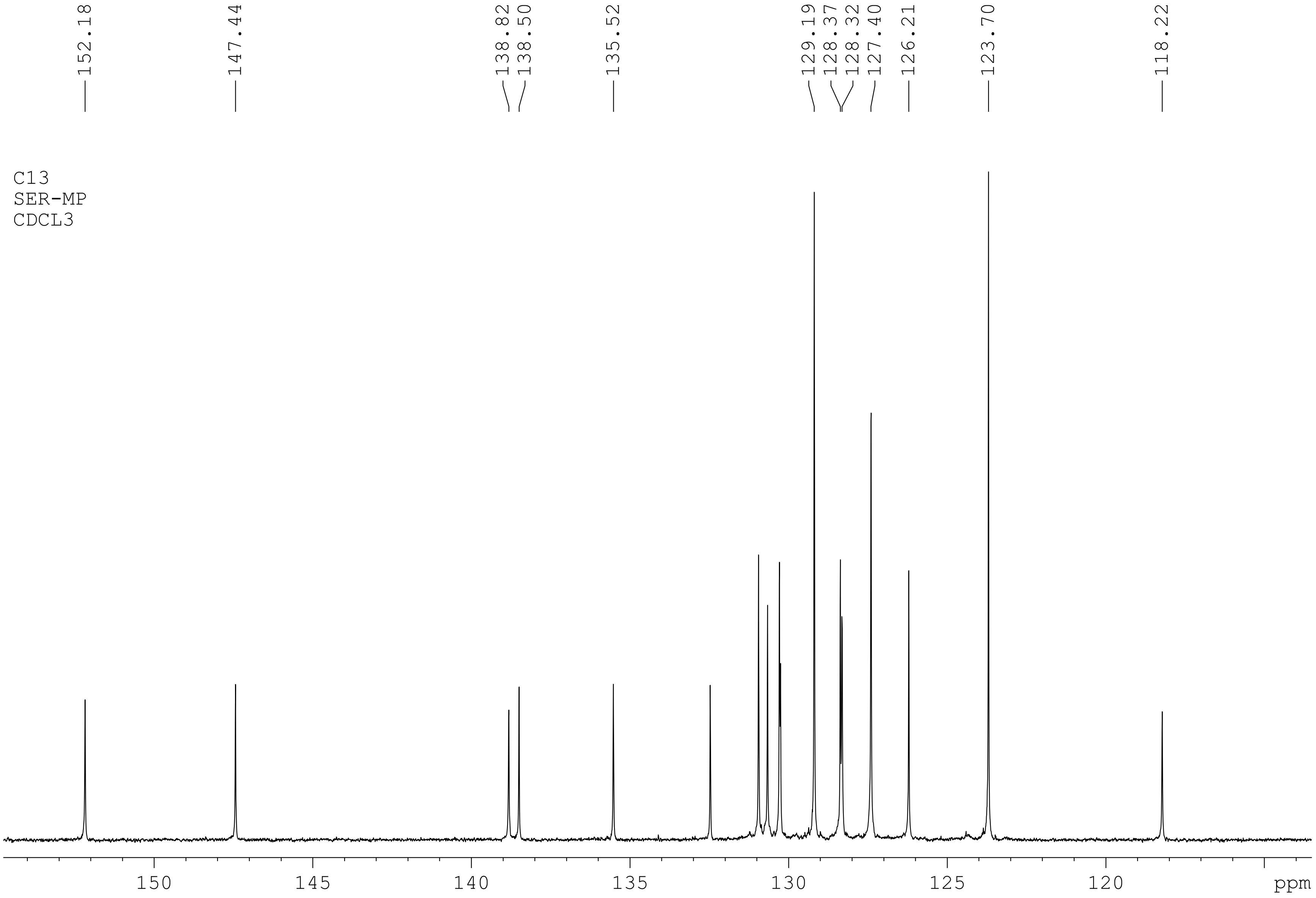
152.18
147.44
138.82
138.50
135.52
129.19
128.37
128.32
127.40
126.21
123.70
118.22
C13
SER-MP
CDCL3
150
145
140
135
130
125
120
ppm

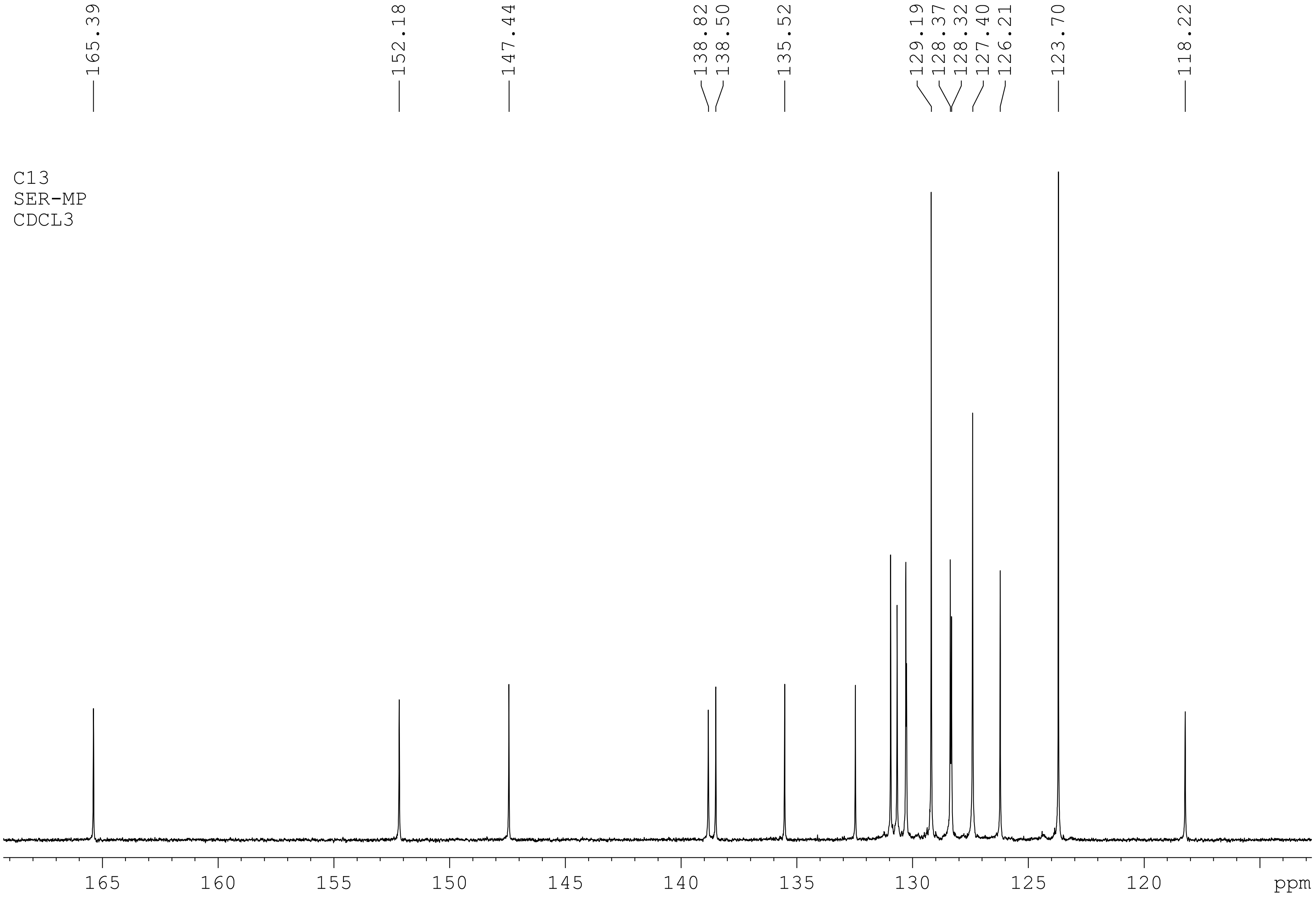
165.39
152.18
147.44
138.82
138.50
135.52
129.19
128.37
128.32
127.40
126.21
123.70
118.22
C13
SER-MP
CDCL3
165
160
155
150
145
140
135
130
125
120
ppm
